# Genetic Variation in 15-Hydroxyprostaglandin Dehydrogenase and Colon Cancer Susceptibility

**DOI:** 10.1371/journal.pone.0064122

**Published:** 2013-05-22

**Authors:** Cheryl L. Thompson, Stephen P. Fink, James D. Lutterbaugh, Robert C. Elston, Martina L. Veigl, Sanford D. Markowitz, Li Li

**Affiliations:** 1 Department of Family Medicine and Community Health, Case Western Reserve University and University Hospitals Case Medical Center, Cleveland, Ohio, United States of America; 2 Department of Epidemiology and Biostatistics, Case Western Reserve University and University Hospitals Case Medical Center, Cleveland, Ohio, United States of America; 3 Department of Medicine, Case Western Reserve University and University Hospitals Case Medical Center, Cleveland, Ohio, United States of America; 4 Case Comprehensive Cancer Center, Case Western Reserve University and University Hospitals Case Medical Center, Cleveland, Ohio, United States of America; MOE Key Laboratory of Environment and Health, School of Public Health, Tongji Medical College, Huazhong University of Science and Technology, China

## Abstract

**Background:**

15-Hydroxyprostaglandin dehydrogenase (15-PGDH) is a metabolic antagonist of COX-2, catalyzing the degradation of inflammation mediator prostaglandin E2 (PGE_2_) and other prostanoids. Recent studies have established the 15-PGDH gene as a colon cancer suppressor.

**Methods:**

We evaluated 15-PDGH as a colon cancer susceptibility locus in a three-stage design. We first genotyped 102 single-nucleotide polymorphisms (SNPs) in the 15-PGDH gene, spanning ∼50 kb up and down-stream of the coding region, in 464 colon cancer cases and 393 population controls. We then genotyped the same SNPs, and also assayed the expression levels of 15-PGDH in colon tissues from 69 independent patients for whom colon tissue and paired germline DNA samples were available. In the final stage 3, we genotyped the 9 most promising SNPs from stages 1 and 2 in an independent sample of 525 cases and 816 controls (stage 3).

**Results:**

In the first two stages, three SNPs (rs1365611, rs6844282 and rs2332897) were statistically significant (p<0.05) in combined analysis of association with risk of colon cancer and of association with 15-PGDH expression, after adjustment for multiple testing. For one additional SNP, rs2555639, the T allele showed increased cancer risk and decreased 15-PGDH expression, but just missed statistical significance (p-adjusted = 0.063). In stage 3, rs2555639 alone showed evidence of association with an odds ratio (TT compared to CC) of 1.50 (95% CI = 1.05–2.15, p = 0.026).

**Conclusions:**

Our data suggest that the rs2555639 T allele is associated with increased risk of colon cancer, and that carriers of this risk allele exhibit decreased expression of 15-PGDH in the colon.

## Introduction

Colon cancer is the end result of a multistep process of genetic and epigenetic changes resulting in the activation of oncogenic pathways as well as inactivation of tumor suppressor pathways [1]. A key event during the progression of colon cancer is the up-regulation of the cyclooxygenase-2 (COX-2) oncogene [2], [3], [4], [5]. COX-2 catalyzes the conversion of arachidonic acid to PGH_2_, which is an intermediate substrate for a variety of bioactive prostaglandins, including PGE_2_ [6], [7], the predominant prostaglandin found in colon cancer tissues [8]. Several lines of evidence suggest that the increased production of PGE_2_ mediates the oncogenic effect of COX-2 [7], [9], [10], [11].

15-Hydroxyprostaglandin dehydrogenase (15-PGDH) is the rate-limiting enzyme in the degradation of prostaglandins, including PGE_2_, and directly antagonizes the COX-2 oncogenic pathway of prostaglandin production [12]. 15-PGDH is highly expressed in normal colon mucosa, is regulated through the TGF-β tumor suppressor pathway, and undergoes loss of expression in colon cancer [13], [14]. We have previously demonstrated the tumor suppressor function of 15-PGDH, finding that re-expression of 15-PGDH in a colon cancer cell line blocks tumor growth following injection into athymic mice, and that knocking out murine 15-PGDH results in an increased development of colon tumors [13], [15]. Moreover, in human studies we found a substantial 12-fold difference in levels of rectal 15-PGDH among individuals with lowest to highest 15-PGDH transcript levels, and that low levels of rectal 15-PGDH were associated with increased colorectal adenoma (a precursor to colon cancer) recurrences [16].

These findings prompted us to examine whether inherited genetic variation at the 15-PGDH locus would explain the wide population variation in levels of colon 15-PGDH, and would also be associated with risk of developing colon cancer. We evaluated the association with colon cancer risk of SNP markers spanning ∼50 kB upstream to ∼40 kB downstream of the 15-PGDH gene coding region in a population-based case-control study in two stages. We then tested these same SNPs for their associations with expression levels of 15-PGDH in colon tissues in a separate patient population.

## Materials and Methods

### Study Design

We employed a 3 stage study design for this project. In the first stage, we investigated all known SNPs in the 15-PGDH gene, spanning 50 kb upstream to 40 kb downstream, for association with risk of colon cancer in a population-based case-control study. In the second stage, we evaluated the association of these same SNPs with 15-PGDH expression in colonic epithelial tissues from an independent sample of patients. We then used a meta-analysis approach to combine the results from stages 1 and 2 in order to identify the most promising SNPs to move forward for stage 3. In stage 3, we genotyped these top SNPs in a second sample of colon cancer patients and population controls for validation.

### Patient Populations

For the association analysis of 15-PGDH SNPs with risk of colon cancer, incident cases were identified from the state of Kentucky Surveillance, Epidemiology and End Results (SEER) registry. Controls were recruited through random digit dialing and friend referrals. The recruitment of this study population was described in more detail earlier [17]. Overall this study population is approximately 94% Caucasian [17]. In order to minimize the effect of population stratification and to increase homogeneity, we limited our analyses to only individuals self-reporting as Caucasians. For the discovery set (stage 1), subjects were recruited from February 2003 through December 2005, and included 464 cases and 393 controls self-reporting as Caucasian. The replication set (used in stage 3) included 525 Caucasian cases and 816 Caucasian controls recruited from January 2006 to June 2010. All participants provided written informed consent, completed an extensive risk factor questionnaire and donated a sample of blood. Whole blood was shipped to the research laboratory at Case Western Reserve University overnight and processed immediately. DNA was isolated from buffy coats separated from whole blood collected in standard ETDA tubes.

To study the effect of SNPs on tissue gene expression (stage 2), normal colon tissue sections were collected from 69 Caucasian patients recruited at University Hospitals Case Medical Center (UHCMC). All participants provided informed consent. RNA and DNA from the tissue samples were prepared by extraction with guanidine isothiocyanate as previously described [18]. Total cellular RNA and genomic DNA were separated by ultracentrifugation of the extract through a cesium cushion. Both studies were approved by the UHCMC institutional review board.

### Genotyping

We included all known SNPs from 50 kb upstream to 40 kb downstream of 15-PGDH that at the time of initiating our study were listed as Illumina Golden Gate validated, and for which ABI TaqMan assays were available. ABI TaqMan chemistry was employed to genotype these samples according to the manufacturer's protocol. Specifically, 2 µl aliquots, containing 5–10 ng of DNA were transferred from 96-well reservoir plates to 384-well assay plates for each individual being genotyped. Multiple 384-well plates were generated; the DNA was dried down, the plates then sealed and frozen until assayed. A 5 µl aliquot of Master Mix, Probe & Primer was robotically added to each well of a 384-well plate previously plated with DNA. PCR [40 Cycles] was carried out on an ABI GeneAmp PCR System 9700 Dual Head Instrument and endpoint reads were carried out using the ABI 7900 Sequence Detection System (SDS). Since TaqMan Chemistry is a PCR-based procedure, all assay mixes were prepared in an amplicon-free room to avoid contamination.

To ensure data quality, each SDS file was individually reviewed before the data were exported to ensure the baseline is properly set. In the 384-well assay layout, the last column of the plate was reserved for water blanks to ensure no contamination occurred during plating. DNA samples, either from Coriell or from our own database, with known genotypes for the SNPs being interrogated in this study were included on each assay plate to serve as positive controls and to identify the 3 genotypes. Four replicate samples were included in the discovery sample (phase 1) and colon tissue expression sample (phase 2), which were genotyped at the same time, and 29 replicate samples were included in the validation sample (phase 3) to confirm accurate genotyping in the study. Genotype concordance of the replicates and control samples was confirmed. If 100% concordance was not observed, the primary data files were reviewed and typically the assay was repeated. The overall call rate was 94.6% (details in Table S1).

### Quantitative Real-Time PCR Measurement of 15-PGDH

Integrity of isolated total RNA was checked using an Agilent 2100 Bioanalyzer (Agilent Technologies, Santa Clara, CA) and concentrations were determined using a ND-1000 Spectrophotometer (NanoDrop, Wilmington, DE). All reverse transcription quantitative real-time PCR assays were performed following the MIQE guidelines [19]. cDNA was synthesized from 1 µg of input RNA using AMV Reverse Transcriptase (Roche, Indianapolis, IN) following the manufactures recommended protocol. Real-time PCR measurement of 15-PGDH was performed using the human hydrolysis Probe/Primer set Hs00168359_m1 (HPGD, NM_000860) from Applied Biosystems (Foster City, CA). A 25 µl reaction mix contained 1 µl (40 ng) of cDNA template and a 1∶20 dilution of an individual primer/probe set in 1X Supermix (Bio-Rad, CA) and was run on a CFX96 optical module (Biorad, Hercules, CA). Thermal cycling conditions for all assays was 95°C for 4 min, followed by 50 cycles of 95°C for 15 sec and 60°C for 1 min. Cytokeratin 20 (KRT20), a marker of colonic epithelial cell mass, was used as the reference gene for normalization and was amplified using the human KRT20 (NM_019010) hydrolysis primer/probe kit Hs00300643_m1 from Applied Biosystems following the same reaction conditions above. KRT20 was selected because it is a specific marker for colonic epithelial mass [7], [15], [16], as well as having uniform expression by microarray analysis across 16 normal colon tissue biopsies and colonic crypt epithelial cells isolated from an additional 5 normal biopsy samples (Markowitz, unpublished data). For each reverse transcription reaction, 15-PGDH and KRT20 quantification cycle (Cq_15-PGDH_ and Cq_KRT20_) values were determined as the average values obtained from three independent real-time PCR reactions. The overall level of 15-PGDH RNA expression was determined as the ratio of 15-PGDH:KRT20 = 2 exp (Cq_15-PGDH_−Cq_KRT20_). RNA that had not undergone the reverse transcriptase step as well as a water sample that was carried through the reverse transcriptase step were used as negative controls and were negative for all assays performed.

### Statistical Analyses

For quality control, each SNP was tested for deviation from Hardy-Weinberg equilibrium (HWE) in the control population via a chi-square test of difference from expectation. SNPs that showed evidence of deviation from HWE (p<0.05) were excluded from further analyses.

Odds ratios (OR) and 95% confidence intervals (CI) for colon cancer were assessed via a logistic regression controlling for age and gender. In the logistic regressions, the allele more common in cases (compared to controls) was considered the risk allele. For each SNP, individuals were coded as 0, 1 or 2, representing the number of risk alleles at that location. The odds ratios were calculated for having one risk allele and for having two risk alleles, compared to having no risk alleles. The overall p-value was calculated for the p-value of the trend for risk per number of risk alleles (additive model)

The difference in mean 15-PGDH tissue expression for each of the three possible genotypes for each SNP was evaluated using a one-way ANOVA with two degrees of freedom. Given that we were testing our *a priori* hypothesis that the risk allele is associated with decreased 15-PGDH expression, we reported one-sided p-values. When the risk allele demonstrated higher expression, a p-value of 1 was assigned.

In order to determine which SNPs showed the most evidence of both association with 15-PGDH expression and risk of colon cancer, we used Fisher's method to combine p-values [20] from the discovery SNP association and expression analyses. Fisher method allows for combining the p-values, especially in multi-stage analysis, to draw similar inference using different statistics calculated from the same samples. Each of the statistics combined tests a different aspect of the biological hypothesis under investigation. Power can be improved by combining the p-values of the different tests. To address multiple testing, we then utilized the false discovery rate (FDR) method of Benjamini and Hochberg [21] to the combined p-values.

The top 9 SNPs identified were then evaluated for association with colon cancer risk in the replication set using the same statistical methods as the discovery set. We combined the results from the first and second case-control samples using a random effect model. All statistics except for the meta-analysis were computed using SAS 9.2 and p-values<0.05 were considered statistically significant.

## Results

### SNP-Colon Cancer Association Discovery

Cases in Phase 1 were more likely to be male and were, on average, older than the controls (Table 1). Of the 102 SNPs evaluated in the discovery population, 25 were either monomorphic or had a MAF <5% in our population. Of the remaining, 2 were found to be out of HWE, and 1 had call rate <80%. These 28 SNPs were excluded from all further analyses. Among the remaining 75 SNPs, 8 were significantly associated with colon cancer risk in the logistic regression model at the p<0.05 (unadjusted for multiple testing) level (Table S1): rs1365611, p<0.0001; rs6844282, p<0.0001; rs2332897, p<0.0001; rs10520282, p = 0.0035; rs1426936, p = 0.012; rs34299544, p = 0.024; rs2555639, p = 0.038; and rs5007089, p = 0.046. All results are given in Table S1, and five of these that met criteria for inclusion in the replication set (based on results in both risk association and the colon tissue gene expression experiment, see also below) are detailed in Table 2.

**Table 1:** Demographics of Case-Control Sample Populations (Phases 1 and 3).

		Phase 1			Phase 3	
	Cases (N = 464)	Controls (N = 393)	p *	Cases (N = 525)	Controls (N = 816)	p*
Gender			<0.0001			<0.0001
Male	203 (51.7)	172 (37.1)		258 (49.1)	287 (35.2)	
Female	190 (48.4)	292 (62.9)		267 (50.9)	529 (64.8)	
Age, mean (SD)	64.6 (10.7)	58.1 (10.9)	<0.0001	63.0 (9.9)	62.5 (9.7)	<0.0001
Age, range	22–89	33–87		31–90	36–90	

* p-value of differences between cases and controls within that phase.

**Table 2:** Association with Colon Cancer of SNPs Selected for Replication in the Discovery Sample (Phase 1).

	Cases (N = 464)	Controls (N = 393)	OR (95% CI)†	p †
rs1365611				**<0.0001**
CC	176 (48.0)	165 (38.9)	4.97 (2.73–9.05)	
CT	174 (47.4)	188 (44.3)	4.59 (2.52–8.33)	
TT	17 (4.6)	71 (16.8)	1.0 (ref)	
rs2253442				0.55
GG	204 (56.4)	231 (52.6)	1.29 (0.71–2.34)	
AG	135 (37.3)	175 (39.9)	1.13 (0.61–2.07)	
AA	23 (6.4)	33 (7.5)	1.0 (ref)	
rs2555639				**0.038**
TT	180 (46.5)	172 (37.5)	1.71 (1.09–2.69)	
CT	163 (42.1)	213 (46.4)	1.28 (0.82–2.01)	
CC	44 (11.4)	74 (16.1)	1.0 (ref)	
rs2555642				0.53
TT	219 (57.1)	245 (54.1)	1.38 (0.75–2.53)	
CT	144 (37.5)	176 (38.9)	1.24 (0.66–2.31)	
CC	21 (5.5)	32 (7.1)	1.0 (ref)	
rs2555622				0.18
AA	158 (42.9)	216 (48.8)	1.0 (ref)	
AC	171 (46.5)	186 (42.0)	0.99 (0.59–1.64)	
CC	39 (10.6)	41 (9.3)	1.31 (0.79–2.17)	
rs6844282				**<0.0001**
CC	132 (34.4)	122 (26.9)	2.38 (1.55–3.67)	
CG	198 (51.6)	211 (46.6)	2.31 (1.55–3.45)	
GG	54 (14.1)	120 (26.5)	1.0 (ref)	
rs11724251				0.053
AA	114 (30.0)	171 (37.5)	1.0 (ref)	
AG	197 (51.8)	208 (45.6)	1.01 (0.68–1.51)	
GG	69 (18.2)	77 (16.9)	1.47 (0.96–2.26)	
rs10019035				0.065
CC	317 (83.0)	374 (82.2)	1.0 (ref)	
CT	64 (16.8)	71 (15.6)	11.9 (1.46–97.5)	
TT	1 (0.3)	10 (2.2)	11.9 (1.49–94.6)	
rs2332897				**<0.0001**
CC	181 (47.0)	175 (38.8)	4.59 (2.60–8.11)	
CA	185 (48.1)	198 (43.9)	4.46 (2.53–7.87)	
AA	19 (4.9)	78 (17.3)	1.0 (ref)	

† Odds ratio (OR) for colon cancer risk and 95% confidence interval (CI) for having one or two risk alleles, compared to having no risk alleles, and the additive model p-value from logistic regression adjusting for age and gender, but not adjusted for multiple testing.

### Association with 15-PGDH Expression

The same complete set of 102 SNPs was evaluated for association with tissue expression levels of 15-PDGH in an independent set of 69 patients (complete results in Table S2). Of these patients, 38 (55%) were male and 31 (45%) were female. The average age was 70.1 (SD = 13.8), and the age range was 18–94. Of the 102 genotyped SNPs, two failed QC and 14 were monomorphic. These were excluded from further analyses. Of the remaining 84 SNPs, four were statistically significantly correlated with expression levels at p<0.05 (Table S2). Detailed expression results are provided for the same 9 SNPs selected for validation (see also below) in Table 3.

**Table 3:** Association to 15-PGDH Colon Expression of Selected SNPs (Phase 2).

	Genotype (N)	Mean (SD) PGDH Expression	p*	p (combined)**	p (FDR adjusted)†
rs1365611	CC (26)	76.3 (32.1)	1	0.0019	0.038
	CT (26)	72.2 (26.1)			
	TT (5)	71.7 (33.2)			
rs2253442	GG (30)	67.5 (28.5)	0.017	0.053	0.10
	AG (30)	86.9 (35.0)			
	AA (2)	84.1 (35.0)			
rs2555639	TT (28)	67.3 (29.4)	0.012	0.0040	0.063
	CT (33)	80.0 (30.6)			
	CC (5)	99.0 (47.1)			
rs2555642	TT (34)	67.6 (28.7)	0.014	0.044	0.10
	CT (30)	86.9 (35.0)			
	CC (3)	84.1 (35.0)			
rs2555622	AA (24)	84.7 (34.9)	0.050	0.051	0.10
	AC (34)	74.9 (33.4)			
	CC (10)	65.8 (20.0)			
rs6844282	CC (19)	79.7 (37.1)	1	0.0019	0.038
	CG (35)	76.2 (30.0)			
	GG (14)	75.6 (34.8)			
rs11724251	AA (19)	92.2 (34.8)	0.045	0.017	0.94
	AG (30)	73.8 (36.1)			
	GG (15)	64.2 (15.3)			
rs10019035	CC (19)	63.2 (17.7)	0.028	0.013	0.11
	CT (6)	80.4 (20.0)			
	TT (0)				
rs2332897	CC (31)	77.7 (34.9)	0.040	0.0008	0.032
	AC (31)	74.1 (26.6)			
	AA (6)	88.1 (49.9)			

* p-value of number of minor alleles (0, 1 or 2; additive model) in linear regression for 15-PGDH expression level in colon mucosa (one-sided).

** Combined p-value of association to 15-PGDH expression and with risk of colon cancer (from logistic regression presented in Table 1) using Fisher's method for combining p-values, unadjusted for multiple testing.

† p-value adjusted for multiple testing using FDR method.

Upon combining the p-values from the expression and association results using Fisher's method, the 9 most statistically significant SNPs (Table 2), were selected for validation by testing for association with colon cancer risk in an independent replication set of cases and controls. Of these top 9 most significant SNPs, 3 (rs1365611, rs6844282 and rs2332897) remained significant after adjustment for multiple testing (Table 3) (rs1365611 p = 0.038, rs6844282 p = 0.038 and rs2332897 p = 0.032), and one more had a multiple testing adjusted p-value of just over 0.05 (rs2555639, p = 0.063).

### Validation Association

In the Phase 3 validation sample, cases were more likely to be male and were older, on average, than the controls, as in Phase 1 (Table 1). The top 9 SNPs, based on the combined p-values showing evidence for association with colon cancer risk and/or 15-PGDH expression in the colon, were selected for validation in the independent set of 525 colon cancer patients and 816 controls. Of these SNPs, rs2555639 demonstrated statistically significant evidence for association with colon cancer risk at the p<0.05 level (via a logistic regression analysis) (Table 4).

**Table 4:** SNP Association Validation Population.

	Cases (N = 525)	Controls (N = 816)	OR (95% CI)†	p †
rs1365611				0.30
CC	244 (47.7)	359 (44.6)	1.14 (0.78–1.66)	
CT	215 (42.0)	358 (44.5)	0.99 (0.67–1.45)	
TT	53 (10.3)	88 (10.9)	1.0 (ref)	
rs2253442				0.13
GG	286 (54.6)	417 (51.4)	1.44 (0.91–2.27)	
AG	207 (39.5)	330 (40.7)	1.32 (0.83–2.10)	
AA	31 (5.9)	64 (7.9)	1.0 (ref)	
rs2555639				0.026
TT	218 (41.8)	303 (37.4)	1.50 (1.05–2.15)	
CT	244 (46.8)	385 (47.5)	1.29 (0.91–1.84)	
CC	59 (11.3)	122 (15.1)	1.0 (ref)	
rs2555642				0.17
TT	285 (54.5)	421 (51.7)	1.42 (0.89–2.28)	
CT	209 (40.0)	233 (40.9)	1.33 (0.82–2.15)	
CC	29 (5.5)	60 (7.4)	1.0 (ref)	
rs2555622				0.15
AA	213 (41.0)	368 (45.3)	1.0 (ref)	
AC	245 (47.1)	358 (44.0)	1.18 (0.93–1.50)	
CC	62 (11.9)	87 (10.7)	1.23 (0.85–1.78)	
rs6844282				0.44
CC	178 (33.9)	239 (29.4)	1.07 (0.78–1.47)	
CG	240 (45.7)	418 (51.5)	0.81 (0.60–1.09)	
GG	107 (20.4)	155 (19.1)	1.0 (ref)	
rs11724251				0.31
AA	161 (30.8)	290 (35.9)	1.0 (ref)	
AG	276 (52.8)	377 (46.6)	1.31 (1.02–1.68)	
GG	86 (16.4)	142 (17.6)	1.10 (0.79–1.53)	
rs10019035				0.85
CC	424 (81.2)	644 (80.5)	1.0 (ref)	
CT	92 (17.6)	148 (18.5)	0.94 (0.71–1.26)	
TT	6 (1.2)	8 (1.0)	1.22 (0.41–3.58)	
rs2332897				0.27
CC	250 (47.7)	360 (44.4)	1.13 (0.78–1.65)	
CA	219 (41.8)	362 (44.6)	0.97 (0.66–1.42)	
AA	55 (10.5)	89 (11.0)	1.0 (ref)	

† Odds ratio (OR) for colon cancer risk, 95% confidence interval (CI) and p-value for trend from logistic regression, with SNP in additive model adjusting for age and gender, but not adjusted for multiple testing.

Combining the data from the case-control discovery and validation populations, our data suggest that having two copies of the T allele of rs2555639 confers an estimated 58% (95%CI: 19%–109%, p = 0.0015) increase in odds of colon cancer compared to individuals with two copies of the C allele (Table 5). Furthermore, the rs2555639 T allele is also associated with decreased expression levels of the 15-PGDH tumor suppressor gene (Table 3, p = 0.012),

**Table 5:** Association of 15-PGDH SNPs with Colon Cancer in Discovery and Validation Populations.

SNP	Discovery OR (95% CI)	Validation OR (95% CI)	Combined OR (95% CI)	Combined p	Heterogeneity p
**rs1365611**	4.97 (2.73–9.05)	1.14 (0.78–1.66)	1.73 (1.26–2.37)	0.0008	0.000045
**rs2253442**	1.29 (0.71–2.34)	1.44 (0.91–2.27)	1.38 (0.96–1.99)	0.079	0.77
**rs2555639**	1.71 (1.09–2.69)	1.50 (1.05–2.15)	1.58 (1.19–2.09)	0.0015	0.66
**rs2555642**	1.38 (0.75–2.53)	1.42 (0.89–2.28)	1.40 (0.97–2.04)	0.074	0.94
**rs2555622**	1.31 (0.79–2.17)	1.23 (0.85–1.78)	1.26 (0.93–1.69)	0.13	0.84
**rs6844282**	2.38 (1.55–3.67)	1.07 (0.78–1.47)	1.42 (1.10–1.83)	0.0078	0.0035
**rs11724251**	1.47 (0.96–2.26)	1.10 (0.79–1.53)	1.22 (0.94–1.59)	0.13	0.29
**rs10019035**	11.9 (1.49–94.6)	1.22 (0.41–3.58)	1.98 (0.76–5.14)	0.16	0.056
**rs2332897**	4.59 (2.60–8.11)	1.13 (0.78–1.65)	1.74 (1.27–2.38)	0.0006	0.000058

## Discussion

**Figure 1:**
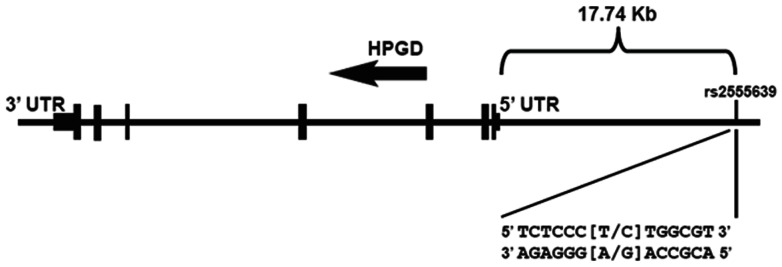
Schematic of HPGD gene, which encodes 15-PGDH, showing location of rs2555639.

Here we present evidence of the association between the T allele of the 15-PGDH rs2555639 SNP and risk of colon cancer in a staged study design. This allele was also associated with decreased 15-PGDH expression in colon tissue in an independent patient population. This SNP maps 17.74 Kb upstream of the 5′ UTR of the 15-PGDH gene, in the presumed regulatory region of the gene (Fig. 1). Our study thus highlights the importance of considering genetic variation in promoter regions when assessing the association of inherited variation with predisposition to disease.

While rs2555639 was the only SNP that was significantly associated with risk of colon cancer in each of the discovery and validation sets independently, several additional SNPs had highly significant association with risk when combining data from the discovery and validation samples (Table 5), including rs1365611 (OR = 1.73, 95%CI:1.26–2.37, p = 0.0008), rs6844282 (OR = 1.42, 95%CI:1.10–1.83, p = 0.0078), and rs2332897 (OR = 1.74, 95%CI:1.27–2.38, p = 0.0006). The discovery and replication sets however show evidence of heterogeneity (Table 5), and these 3 SNPs were not significant in the validation sample. Further study with a larger sample size will be required before any final conclusions can be reached regarding the association of these three additional SNPs with risk of colon cancer.

**Figure 2:**
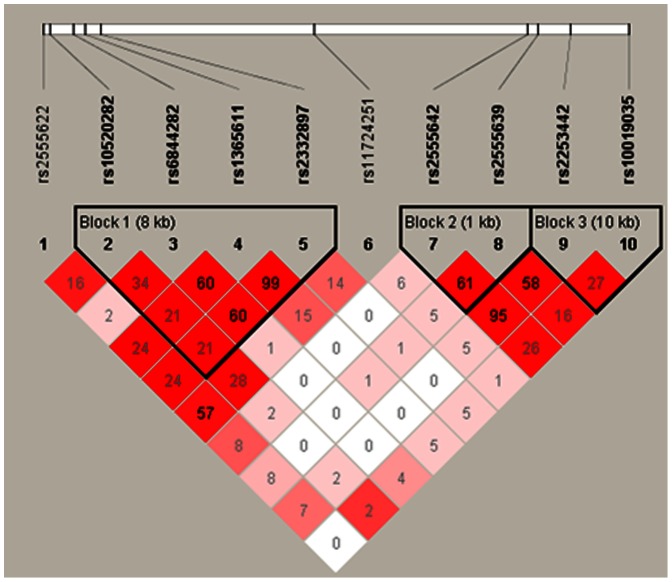
Linkage Disequilibrium (LD) Plot of Genotyped SNPs. LD plot of all SNPs selected for replication in all Caucasian samples. Values within boxes are correlations (R^2^).

SNP rs2555639 falls into an extremely small LD block, with poor correlations with neighboring SNPs. This may explain why no other SNPs in the tagging panel we tested were significantly associated with disease risk (Fig. 2). For this same reason, rs2555639 would be unlikely to have been detected in previous genome-wide association studies (GWAS) that relied on selections of panels of tagging SNPs, and did not find a statistically significant association in the 15-PGDH region [22], [23], [24].

Similarly, previous candidate gene studies of the association of the 15-PGDH SNPs with colon cancer risk also failed to detect rs2555639. These earlier studies identified two SNPs in PGDH – rs2612656 and rs8752 – as individually showing significant association with colon cancer risk [25]. However, neither was replicated in a validation study [26]. Both these two previous studies limited the region examined to either the body of the 15-PGDH gene or to only 5 kb of flanking genomic sequence [25], [26]. Thus, neither of these studies would have detected the association of rs2555639 with colon cancer risk. Another earlier study evaluated the association of only two non-synonymous coding SNPs in 15-PGDH with colon adenoma risk [27]. We did not evaluate the association of these SNPs with risk of colon cancer in our study because of their low minor allele frequencies (3% and 1%, respectively).

One limitation of our study is that we only evaluated the association of 15-PGDH locus SNPs with colon cancer risk among individuals self-reporting as Caucasian, who are predominantly of European ancestry. Thus we are unable to evaluate whether the association of rs2555639 with both colon cancer risk and 15-PGDH expression holds in other racial groups. In addition, we excluded SNPs with a minor allele frequency less than 5%. This, in combination with our relatively small discovery sample size, may have limited our ability to detect any association to either rare 15-PGDH variants or to more common variants with very low effects. Another potential limitation is that both our stage 1 and stage 3 samples were drawn from the State of Kentucky population. Validation of our results in other independent, non-Kentuckian populations is thus warranted.

The important role of COX-2 and the arachidonic acid pathway in the development of colon cancer is well established, as is the role of 15-PGDH as a metabolic suppressor of the COX-2 pathway and a colon cancer suppressor gene [4], [5]. In this study we have demonstrated evidence for inherited variations in the 15-PGDH gene in potentially regulating 15-PGDH expression levels in the colon as well as conferring susceptibility to colon cancer. We have identified a single SNP, rs2555639, 17.74 kb upstream of the 5′ UTR of the 15-PGDH gene, which is associated both with lower colonic 15-PGDH expression and with increased risk of colon cancer. This study illustrates the advantage of combining tests of SNP association with tissue 15-PGDH expression and with disease risk, as this combined approach has allowed us to identify the 15-PGDH rs2555639 T allele as a potentially functional and novel colon cancer susceptibility variant in a 3-stage study despite the modest sample sizes of both the discovery and replication case-control sets. We shall point out that we used α = 0.05 as the cut-off to declare replication significance in the validation phase without further adjustment for multiple testing. Although the validation SNPs were selected based on the combined evidence from stages 1 and 2 for their association with both risk of colon cancer and 15-PGDH tissue expression, caution must be taken in interpreting our replication results. Nevertheless, our results should stimulate further studies to validate the rs25556399 variant as predisposing to colon cancer in other independent populations, as well as to investigate other SNP variants in the 15-PGDH locus in the development of colon cancer.

## Supporting Information

Table S1 **Complete SNP-Colon Cancer Association Results in Discovery Population.** Distribution (N(%))of homozygous major allele, heterozygous and homozygous minor allele, and OR of homozygous risk (defined as more common in cases compared to controls) vs. homozygous reference.
(DOCX)

Table S2 **Complete SNP-Expression Results in Tissue Sample Population.**
(DOCX)
