# Supplementary material for: Genetic Variation in 15-Hydroxyprostaglandin Dehydrogenase and Colon Cancer Susceptibility
Source: PLoS One. 2013 May 22;8(5):e64122. doi: 10.1371/journal.pone.0064122 (PMC3661460; doi:10.1371/journal.pone.0064122)
Supplement: Table S1 — Complete SNP-Colon Cancer Association Results in Discovery Population. Distribution (N(%))of homozygous major allele, heterozygous and homozygous minor allele, and OR of homozygous risk (defined as more common in cases compared to controls) vs. homozygous reference. (DOCX) [file pone.0064122.s001.docx]

**Table S1: Complete SNP-Colon Cancer Association Results in Discovery Population**

|  | **Cases (N=464)** | **Controls (N=393)** | **OR (95% CI)*** | **p*** |
| --- | --- | --- | --- | --- |
| Gender |  |  |  |  |
| Male | 203 (51.7) | 172 (37.1) |  |  |
| Female | 190 (48.4) | 292 (62.9) |  |  |
| Age | 64.6 (10.7) | 58.1 (10.9) |  |  |
| rs1035804 | 169 (44.5) | 221 (48.6) | 1.19 (0.94-1.51) | 0.15 |
|  | 178 (46.8) | 194 (42.6) |  |  |
|  | 33 (8.7) | 40 (8.8) |  |  |
| rs2555622 | 158 (42.9) | 216 (48.8) | 1.31 (0.79-2.17) | 0.18 |
|  | 171 (46.5) | 186 (42.0) |  |  |
|  | 39 (10.6) | 41 (9.3) |  |  |
| rs2612663 | 171 (44.4) | 220 (48.5) | 1.22 (0.96-1.54) | 0.1 |
|  | 180 (46.8) | 195 (43.0) |  |  |
|  | 34 (8.8) | 39 (8.6) |  |  |
| rs10520282 | 160 (44.0) | 246 (56.7) | 1.45 (0.84-2.49) | 0.0035 |
|  | 170 (46.7) | 154 (35.5) |  |  |
|  | 34 (9.3) | 34 (7.8) |  |  |
| rs2612665 | 186 (62.5) | 228 (65.5) | 1.09 (0.46-2.58) | 0.63 |
|  | 101 (33.9) | 107 (30.8) |  |  |
|  | 11 (3.7) | 13 (3.7) |  |  |
| rs2555626 | 341 (90.5) | 400 (89.3) | Low MAF |  |
|  | 36 (9.6) | 48 (10.7) |  |  |
|  | 0 (0) | 0 (0) |  |  |
| rs2612666 | 348 (89.9) | 411 (89.2) | Low MAF |  |
|  | 37 (9.6) | 49 (10.6) |  |  |
|  | 2 (0.5) | 1 (0.2) |  |  |
| rs6844282 | 132 (13.4) | 122 (26.9) | 2.38 (1.55-3.67) | <0.0001 |
|  | 198 (52.0) | 211 (46.6) |  |  |
|  | 54 (13.4) | 120 (26.5) |  |  |
| rs17052152 | 146 (45.5) | 219 (57.9) | 1.29 (0.90-1.67) | 0.06 |
|  | 150 (46.7) | 134 (35.5) |  |  |
|  | 25 (7.8) | 25 (6.6) |  |  |
| rs2195039 | 292 (80.7) | 349 (80.8) | 1.18 (0.83-1.68) | 0.36 |
|  | 65 (18.0) | 76 (17.6) |  |  |
|  | 5 (1.4) | 7 (1.6) |  |  |
| rs1365611 | 176 (48.0) | 165 (38.9) | 4.97 (2.73-9.05) | <0.0001 |
|  | 174 (47.4) | 188 (44.3) |  |  |
|  | 17 (4.6) | 71 (16.8) |  |  |
| rs1426936 | 110 (30.4) | 183 (43.0) | 1.33 (1.06-1.67) | 0.012 |
|  | 192 (53.0) | 179 (42.0) |  |  |
|  | 60 (16.6) | 64 (15.0) |  |  |
| rs2332897 | 181 (47.0) | 175 (38.8) | 4.59 (2.60-8.11) | <0.0001 |
|  | 185 (48.1) | 198 (43.9) |  |  |
|  | 19 (4.9) | 78 (17.3) |  |  |
| rs17052161 | 310 (81.6) | 364 (82.5) | 1.20 (0.85-1.68) | 0.31 |
|  | 63 (16.6) | 68 (15.4) |  |  |
|  | 7 (1.8) | 9 (2.0) |  |  |
| rs17359173 | 355 (92.0) | 427 (93.4) | Low MAF |  |
|  | 30 (7.8) | 29 (6.4) |  |  |
|  | 1 (0.3) | 1 (0.2) |  |  |
| rs3846297 | 268 (70.3) | 335 (74.1) | 1.25 (0.93-1.69) | 0.15 |
|  | 104 (27.3) | 110 (24.3) |  |  |
|  | 9 (2.4) | 7 (1.6) |  |  |
| rs7680964 | 382 (99.5) | 453 (98.9) | Low MAF |  |
|  | 2 (0.5) | 5 (1.1) |  |  |
|  | 0 (0) | 0 (0) |  |  |
| rs6827776 | 312 (81.0) | 363 (80.1) | 0.91 (0.64-1.28) | 0.58 |
|  | 69 (17.9) | 83 (18.3) |  |  |
|  | 4 (1.0) | 7 (1.6) |  |  |
| rs10520284 | 345 (92.3) | 417 (93.9) | Low MAF |  |
|  | 29 (7.8) | 26 (5.9) |  |  |
|  | 0 (0) | 1 (0.2) |  |  |
| rs4147100 | 300 (78.5) | 350 (79.4) | 1.30 (0.93-1.81) | 0.12 |
|  | 74 (19.4) | 84 (19.1) |  |  |
|  | 8 (2.1) | 7 (1.6) |  |  |
| rs17359381 | 335 (90.5) | 415 (93.7) | Low MAF |  |
|  | 34 (9.2) | 28 (6.3) |  |  |
|  | 1 (0.3) | 0 (0) |  |  |
| rs2098948 | 239 (61.4) | 293 (65.4) | 1.17 (0.90-1.53 | 0.24 |
|  | 130 (33.4) | 141 (31.5) |  |  |
|  | 20 (5.1) | 14 (3.1) |  |  |
| rs4613543 | 347 (91.6) | 423 (93.2) | Low MAF |  |
|  | 32 (8.4) | 30 (6.6) |  |  |
|  | 0 (0) | 1 (0.2) |  |  |
| rs4496559 | 384 (100) | 454 (100) | Low MAF |  |
|  | 0 (0) | 0 (0) |  |  |
|  | 0 (0) | 0 (0) |  |  |
| rs17060521 | 289 (74.7) | 343 (75.4) | 1.00 (0.76-1.35) | 0.98 |
|  | 88 (22.7) | 100 (22.0) |  |  |
|  | 10 (2.6) | 12 (2.6) |  |  |
| rs10520285 | 355 (92.7) | 430 (94.1) | Low MAF |  |
|  | 28 (7.3) | 26 (5.7) |  |  |
|  | 0 (0) | 1 (0.2) |  |  |
| rs2253270 | 371 (96.6) | 449 (98.3) | Low MAF |  |
|  | 12 (3.1) | 7 (1.5) |  |  |
|  | 1 (0.3) | 1 (0.2) |  |  |
| rs2253170 | 379 (98.4) | 456 (99.1) | Low MAF |  |
|  | 6 (1.6) | 4 (0.9) |  |  |
|  | 0 (0) | 0 (0) |  |  |
| rs13126570 | 278 (72.4) | 337 (74.6) | 1.34 (0.99-1.80) | 0.06 |
|  | 94 (24.5) | 104 (23.0) |  |  |
|  | 12 (3.1) | 11 (2.4) |  |  |
| rs34299544 | 278 (72.8) | 338 (76.1) | Not in HWE | 0.024 |
|  | 90 (23.8) | 94 (21.2) |  |  |
|  | 13 (3.4) | 12 (2.7) |  |  |
| rs2555661 | 377 (98.4) | 451 (99.1) | Low MAF |  |
|  | 6 (1.6) | 4 (0.9) |  |  |
|  | 0 (0) | 0 (0) |  |  |
| rs6419992 | 218 (57.5) | 283 (62.3) | 1.21 (0.93-1.57) | 0.15 |
|  | 144 (38.0) | 151 (33.4) |  |  |
|  | 17 (4.5) | 20 (4.4) |  |  |
| rs17360116 | 337 (92.3) | 399 (93.9) | Low MAF |  |
|  | 28 (7.7) | 26 (6.1) |  |  |
|  | 0 (0) | 0 (0) |  |  |
| rs11724251 | 114 (30.0) | 171 (37.5) | 1.47 (0.96-2.26) | 0.053 |
|  | 197 (51.8) | 208 (45.6) |  |  |
|  | 69 (18.2) | 77 (16.9) |  |  |
| rs17060554 | 287 (74.4) | 339 (75.2) | 1.28 (0.95-1.72) | 0.11 |
|  | 86 (22.3) | 100 (22.2) |  |  |
|  | 13 (3.4) | 12 (2.7) |  |  |
| rs7349744 | 134 (34.9) | 140 (31.0) | 1.08 (0.87-1.33) | 0.48 |
|  | 173 (45.1) | 215 (47.6) |  |  |
|  | 77 (20.1) | 97 (21.5) |  |  |
| rs17060557 | 347 (91.3) | 415 (93.3) | Low MAF | 0.58 |
|  | 22 (8.4) | 28 (6.3) |  |  |
|  | 1 (0.3) | 2 (0.5) |  |  |
| rs2555630 | 375 (97.7) | 450 (97.8) | Low MAF |  |
|  | 8 (2.1) | 10 (2.2) |  |  |
|  | 1 (0.3) | 0 (0) |  |  |
| rs2555631 | 357 (97.8) | 428 (97.7) | Low MAF |  |
|  | 6 (1.6) | 10 (2.3) |  |  |
|  | 2 (0.6) | 0 (0) |  |  |
| rs3737012 | 326 (84.5) | 369 (81.8) | Low MAF |  |
|  | 60 (15.5) | 78 (17.3) |  |  |
|  | 0 (0) | 4 (0.9) |  |  |
| rs2254350 | 366 (97.1) | 447 (97.8) | Low MAF |  |
|  | 9 (2.4) | 10 (2.2) |  |  |
|  | 2 (0.5) | 0 (0) |  |  |
| rs2612660 | 371 (97.6) | 440 (97.8) | Low MAF |  |
|  | 9 (2.4) | 10 (2.2) |  |  |
| rs12644138 | 114 (72.2) | 117 (62.9) | Low Call Rate |  |
|  | 37 (23.4) | 65 (34.9) |  |  |
|  | 7 (4.4) | 4 (2.2) |  |  |
| rs12500316 | 200 (51.8) | 263 (58.1) | 1.16 (0.91-1.49) | 0.23 |
|  | 157 (40.7) | 167 (36.9) |  |  |
|  | 29 (7.5) | 23 (5.1) |  |  |
| rs17060596 | 317 (83.6) | 354 (81.4) | 1.38 (0.95-2.01) | 0.089 |
|  | 60 (15.8) | 75 (17.2) |  |  |
|  | 2 (0.5) | 6 (1.4) |  |  |
| rs3797009 | 266 (70.0) | 299 (65.7) | 1.19 (0.90-1.57) | 0.22 |
|  | 100 (26.3) | 139 (30.6) |  |  |
|  | 14 (3.7) | 17 (3.7) |  |  |
| rs1365614 |  |  | Low MAF |  |
| rs1050145 | 119 (31.6) | 158 (35.0) | 1.14 (0.93-1.41) | 0.21 |
|  | 172 (45.6) | 204 (45.2) |  |  |
|  | 86 (22.8) | 89 (19.7) |  |  |
| rs1346270 | 386 (100) | 455 (100) | Low MAF |  |
| rs6825010 | 375 (100) | 449 (100) | Low MAF |  |
| rs1426945 | 137 (35.7) | 151 (33.1) | 1.19 (0.96-1.46) | 0.11 |
|  | 180 (46.9) | 199 (43.6) |  |  |
|  | 67 (17.5) | 106 (23.3) |  |  |
| rs12505520 | 384 (100) | 453 (100) | Low MAF |  |
| rs3756273 | 124 (32.9) | 153 (33.7) | 1.01 (0.82-1.24) | 0.94 |
|  | 183 (47.8) | 201 (44.3) |  |  |
|  | 76 (19.8) | 100 (22.0) |  |  |
| rs4147098 | 139 (36.5) | 182 (40.3) | 1.11 (0.89-1.37) | 0.36 |
|  | 181 (47.5) | 195 (43.1) |  |  |
|  | 61 (16.0) | 75 (16.6) |  |  |
| rs1834693 | 260 (68.6) | 321 (70.9) | 0.90 (0.68-1.18) | 0.45 |
|  | 107 (28.2) | 115 (25.4) |  |  |
|  | 12 (3.2) | 17 (3.8) |  |  |
| rs13108146 | 233 (60.5) | 257 (56.1) | 1.12 (0.86-1.45) | 0.41 |
|  | 134 (34.8) | 177 (38.7) |  |  |
|  | 18 (4.7) | 24 (5.2) |  |  |
| rs17361009 | 338 (88.3) | 368 (81.8) | 1.40 (0.93-2.11) | 0.11 |
|  | 43 (11.2) | 80 (17.8) |  |  |
|  | 2 (0.5) | 2 (0.4) |  |  |
| rs5007089 | 198 (52.7) | 192 (42.5) | 0.79 (0.62-1.00) | 0.046 |
|  | 141 (37.5) | 225 (49.8) |  |  |
|  | 37 (9.8) | 35 (7.7) |  |  |
| rs2255577 | 380 (99.0) | 453 (99.6) | Low MAF |  |
|  | 4 (1.0) | 2 (0.4) |  |  |
|  | 0 (0) | 0 (0) |  |  |
| rs12645863 | 280 (73.3) | 333 (73.7) | 1.03 (0.75-1.40) | 0.88 |
|  | 95 (24.9) | 109 (24.1) |  |  |
|  | 7 (1.8) | 10 (2.2) |  |  |
| rs2251316 | 191 (49.2) | 225 (49.7) | 1.04 (0.82-1.31) | 0.75 |
|  | 159 (41.0) | 190 (41.9) |  |  |
|  | 38 (9.8) | 38 (8.4) |  |  |
| rs17295290 | 281 (73.2) | 318 (70.5) | 1.05 (0.78-1.41) | 0.76 |
|  | 91 (23.7) | 123 (27.3) |  |  |
|  | 12 (3.1) | 10 (2.2) |  |  |
| rs2555643 | 217 (57.3) | 244 (54.1) | 1.22 (0.95-1.56) | 0.12 |
|  | 141 (37.2) | 176 (39.0) |  |  |
|  | 21 (5.5) | 31 (6.9) |  |  |
| rs2555642 | 219 (57.1) | 245 (54.1) | 1.38 (0.75-2.53) | 0.53 |
|  | 144 (37.5) | 176 (38.9) |  |  |
|  | 21 (5.5) | 32 (7.1) |  |  |
| rs12643132 | 277 (73.1) | 333 (75.2) | 0.89 (0.65-1.22) | 0.47 |
|  | 95 (25.1) | 101 (22.8) |  |  |
|  | 7 (1.9) | 9 (2.0) |  |  |
| rs7657391 | 247 (64.7) | 309 (67.6) | 1.18 (0.91-1.55) | 0.22 |
|  | 118 (30.9) | 134 (29.3) |  |  |
|  | 17 (4.5) | 14 (3.1) |  |  |
| rs17361254 | 280 (73.3) | 320 (70.6) | 0.96 (0.71-1.29) | 0.76 |
|  | 90 (23.6) | 124 (27.4) |  |  |
|  | 12 (3.1) | 9 (2.0) |  |  |
| rs12644005 | 285 (73.6) | 333 (73.8) | 0.98 (0.72-1.34) | 0.9 |
|  | 95 (24.6) | 108 (24.0) |  |  |
|  | 7 (1.8) | 10 (2.2) |  |  |
| rs1346269 | 326 (83.8) | 349 (76.4) | 1.40 (0.98-1.99) | 0.066 |
|  | 59 (15.2) | 103 (22.5) |  |  |
|  | 4 (1.0) | 5 (1.1) |  |  |
| rs17295603 | 321 (83.8) | 342 (76.7) | 1.37 (0.96-1.96) | 0.085 |
|  | 58 (15.1) | 99 (22.2) |  |  |
|  | 4 (1.0) | 5 (1.1) |  |  |
| rs2555639 | 180 (46.5) | 172 (37.5) | 1.71 (1.09-2.69) | 0.038 |
|  | 163 (42.1) | 213 (46.4) |  |  |
|  | 44 (11.4) | 74 (16.1) |  |  |
| rs11725947 | 280 (73.5) | 320 (71.3) | 0.96 (0.71-1.29) | 0.77 |
|  | 89 (23.4) | 119 (26.5) |  |  |
|  | 12 (3.2) | 10 (2.2) |  |  |
| rs2256673 | 278 (72.4) | 323 (71.3) | 1.00 (0.74-1.34) | 0.98 |
|  | 94 (24.5) | 120 (26.5) |  |  |
|  | 12 (3.1) | 10 (2.2) |  |  |
| rs2256669 | 257 (66.8) | 315 (69.2) | 0.91 (0.70-1.20) | 0.51 |
|  | 115 (29.9) | 122 (26.8) |  |  |
|  | 13 (3.4) | 18 (4.0) |  |  |
| rs11133044 | 213 (55.9) | 270 (59.2) | 1.14 (0.88-1.48) | 0.33 |
|  | 147 (38.6) | 167 (36.6) |  |  |
|  | 21 (5.5) | 19 (4.2) |  |  |
| rs2555674 | 214 (56.3) | 243 (55.2) | 1.17 (0.91-1.49) | 0.22 |
|  | 143 (37.6) | 164 (37.3) |  |  |
|  | 23 (6.1) | 33 (7.5) |  |  |
| rs6811079 | 278 (82.0) | 325 (82.1) | 1.31 (0.89-1.91) | 0.17 |
|  | 60 (17.7) | 64 (16.2) |  |  |
|  | 1 (0.3) | 7 (1.8) |  |  |
| rs2253442 | 204 (56.4) | 231 (52.6) | 1.29 (0.71-2.34) | 0.55 |
|  | 135 (37.3) | 175 (39.9) |  |  |
|  | 23 (6.4) | 33 (7.5) |  |  |
| rs2042755 | 140 (36.8) | 192 (42.2) | 1.24 (0.99-1.56) | 0.062 |
|  | 190 (50.0) | 213 (46.8) |  |  |
|  | 50 (13.2) | 50 (11.0) |  |  |
| rs2042756 | 142 (38.2) | 191 (42.2) | 1.20 (0.96-1.50) | 0.11 |
|  | 180 (48.4) | 209 (46.1) |  |  |
|  | 50 (13.4) | 53 (11.7) |  |  |
| rs1820526 | 222 (61.0) | 274 (63.9) | 1.12 (0.86-1.47) | 0.41 |
|  | 123 (33.8) | 136 (31.7) |  |  |
|  | 19 (5.2) | 19 (4.4) |  |  |
| rs1365625 | 141 (38.4) | 192 (44.3) | 1.24 (0.99-1.54) | 0.061 |
|  | 172 (46.9) | 184 (42.5) |  |  |
|  | 54 (14.7) | 57 (13.2) |  |  |
| rs2612677 | 252 (65.8) | 310 (68.6) | 0.90 (0.69-1.18) | 0.46 |
|  | 118 (30.8) | 124 (27.4) |  |  |
|  | 13 (3.4) | 18 (4.0) |  |  |
| rs17060632 | 286 (75.1) | 310 (70.3) | 0.86 (0.63-1.17) | 0.34 |
|  | 85 (22.3) | 124 (28.1) |  |  |
|  | 10 (2.6) | 7 (1.6) |  |  |
| rs2877818 | 313 (82.8) | 363 (82.3) | 1.34 (0.94-1.91) | 0.11 |
|  | 64 (16.9) | 69 (15.7) |  |  |
|  | 1 (0.3) | 9 (2.0) |  |  |
| rs10520286 | 234 (61.7) | 268 (62.0) | 1.06 (0.81-1.39) | 0.67 |
|  | 128 (33.8) | 153 (35.4) |  |  |
|  | 17 (4.5) | 11 (2.6) |  |  |
| rs10019035 | 317 (83.0) | 374 (82.2) | 11.9 (1.49-94.6) | 0.065 |
|  | 64 (16.8) | 71 (15.6) |  |  |
|  | 1 (0.3) | 10 (2.2) |  |  |
| rs10032848 | 119 (31.1) | 121 (27.5) | 1.15 (0.93-1.43) | 0.2 |
|  | 188 (49.1) | 228 (51.8) |  |  |
|  | 76 (19.8) | 91 (20.7) |  |  |
| rs10019203 | 118 (31.1) | 123 (28.2) | 1.15 (0.92-1.43) | 0.22 |
|  | 186 (49.1) | 223 (51.0) |  |  |
|  | 75 (19.8) | 91 (20.8) |  |  |
| rs9998916 | 304 (82.8) | 332 (82.6) | 1.36 (0.94-1.96) | 0.11 |
|  | 62 (16.9) | 62 (15.4) |  |  |
|  | 1 (0.3) | 8 (2.0) |  |  |
| rs1978567 |  |  | Low MAF |  |
| rs17060646 | 119 (30.8) | 120 (26.6) | 1.16 (0.94-1.44) | 0.16 |
|  | 184 (47.7) | 233 (51.7) |  |  |
|  | 83 (21.5) | 98 (21.7) |  |  |
| rs10520281 | 314 (82.9) | 373 (82.3) | 1.32 (0.93-1.88) | 0.12 |
|  | 64 (16.9) | 70 (15.5) |  |  |
|  | 1 (0.3) | 10 (2.2) |  |  |
| rs2332893 | 291 (76.0) | 341 (76.3) | 0.98 (0.72-1.32) | 0.88 |
|  | 82 (21.4) | 93 (20.8) |  |  |
|  | 10 (2.6) | 13 (2.9) |  |  |
| rs17362255 | 224 (57.7) | 254 (55.8) | 1.21 (0.94-1.55) | 0.14 |
|  | 146 (37.6) | 171 (37.6) |  |  |
|  | 18 (4.5) | 30 (6.6) |  |  |
| rs17296454 | 166 (43.9) | 174 (39.6) | 0.97 (0.78-1.21) | 0.8 |
|  | 158 (41.8) | 208 (47.4) |  |  |
|  | 54 (14.3) | 57 (13.0) |  |  |
| rs7657168 | 173 (51.0) | 202 (53.4) | 1.03 (0.80-1.31) | 0.84 |
|  | 138 (40.7) | 136 (36.0) |  |  |
|  | 28 (8.3) | 40 (10.6) |  |  |
| rs7687916 | 164 (43.4) | 173 (38.6) | 0.95 (0.76-1.18) | 0.63 |
|  | 159 (42.1) | 216 (48.2) |  |  |
|  | 55 (14.6) | 59 (13.2) |  |  |
| rs1365621 | 126 (32.8) | 153 (34.2) | 1.06 (0.85-1.31) | 0.61 |
|  | 188 (49.0) | 205 (45.8) |  |  |
|  | 70 (18.2) | 90 (20.1) |  |  |
| rs12646791 | 283 (73.7) | 329 (73.0) | 1.10 (0.82-1.49) | 0.51 |
|  | 91 (23.7) | 110 (24.4) |  |  |
|  | 10 (2.6) | 12 (2.7) |  |  |
| rs17362542 | 301 (77.6) | 368 (81.4) | 0.87 (0.61-1.23) | 0.41 |
|  | 84 (21.7) | 80 (17.7) |  |  |
|  | 3 (0.8) | 4 (0.9) |  |  |
| rs3113888 | 110 (28.7) | 122 (27.0) | 1.00 (0.81-1.23) | 0.97 |
|  | 175 (45.6) | 229 (50.7) |  |  |
|  | 99 (25.8) | 101 (22.4) |  |  |

*OR (95% CI) and p-value for logistic regression of SNP in additive model for association with colon cancer risk
