# Supplementary material for: Genetic Variation in 15-Hydroxyprostaglandin Dehydrogenase and Colon Cancer Susceptibility
Source: PLoS One. 2013 May 22;8(5):e64122. doi: 10.1371/journal.pone.0064122 (PMC3661460; doi:10.1371/journal.pone.0064122)
Supplement: Table S2 — Complete SNP-Expression Results in Tissue Sample Population. (DOCX) [file pone.0064122.s002.docx]

**Table S2: Complete SNP-Expression Results in Tissue Sample Population**

|  | **Genotype (N)** | **Mean (SD) PGDH Expression** | **p*** |
| --- | --- | --- | --- |
| rs1035804 | CC (24) | 84.7 (34.9) | 0.44 |
|  | CT (33) | 74.4 (34.7) |  |
|  | TT (7) | 70.7 (17.8) |  |
| rs2555622 | AA (24) | 84.7 (34.9) | 0.27 |
|  | AC (34) | 74.9 (33.4) |  |
|  | CC (10) | 65.8 (20.0) |  |
| rs2612663 | GG (23) | 84.9 (35.7) | 0.37 |
|  | AG (34) | 73.8 (34.4) |  |
|  | AA (9) | 70.1 (15.6) |  |
| rs10520282 | TT (30) | 76.2 (30.7) | 0.094 |
|  | CT (24) | 80.4 (36.0) |  |
|  | CC (3) | 120.4 (25.5) |  |
| rs2612665 | AA (34) | 79.0 (33.4) | 0.66 |
|  | AG (24) | 71.4 (34.4) |  |
|  | GG (3) | 82.7 (18.6) |  |
| rs2555626 | AA (49) | 74.2 (27.6) | 0.51 |
|  | AG (8) | 67.2 (26.2) |  |
|  | GG (0) |  |  |
| rs2612666 | TT (58) | 77.2 (31.7) | 0.90 |
|  | TC (10) | 75.8 (39.5) |  |
|  | CC (0) |  |  |
| rs6844282 | CC (19) | 79.7 (37.1) | 0.92 |
|  | CG (35) | 76.2 (30.0) |  |
|  | GG (14) | 75.6 (34.8) |  |
| rs17052152 | CC (32) | 70.6 (28.7) | 0.095 |
|  | CG (28) | 77.9 (35.3) |  |
|  | GG (4) | 107.6 (33.1) |  |
| rs2195039 | AA (47) | 78.6 (34.7) | 0.33 |
|  | AG (19) | 69.9 (26.6) |  |
|  | GG (0) |  |  |
| rs1365611 | CC (26) | 76.3 (32.1) | 0.87 |
|  | CT (26) | 72.2 (26.1) |  |
|  | TT (5) | 71.7 (33.2) |  |
| rs1426936 | AA (21) | 79.6 (35.4) | 0.053 |
|  | AG (36) | 69.7 (28.4) |  |
|  | GG (10) | 97.4 (36.3) |  |
| rs2332897 | CC (31) | 77.7 (34.9) | 0.63 |
|  | AC (31) | 74.1 (26.6) |  |
|  | AA (6) | 88.1 (49.9) |  |
| rs17052161 | AA (45) | 79.1 (34.5) | 0.48 |
|  | AG (19) | 72.8 (26.5) |  |
|  | GG (0) |  |  |
| rs17359173 | GG (63) | 78.4 (33.0) | 0.27 |
|  | AG (3) | 56.7 (26.3) |  |
|  | AA (0) |  |  |
| rs3846297 | CC (45) | 80.1 (35.5) | 0.39 |
|  | CT (20) | 69.5 (26.7) |  |
|  | TT (1) | 100.0 (-) |  |
| rs7680964 | CC (65) | 75.3 (31.9) | N/A |
| rs6827776 | GG (47) | 79.4 (35.2) | 0.36 |
|  | AG (20) | 71.3 (26.7) |  |
|  | AA (0) |  |  |
| rs10520284 | AA (64) | 77.6 (32.9) | 0.28 |
|  | AG (3) | 56.7 (26.3) |  |
|  | GG (0) |  |  |
| rs4147100 | GG (43) | 80.5 (34.7) | 0.33 |
|  | AG (20) | 71.6 (29.7) |  |
|  | AA (0) |  |  |
| rs17359381 | GG (64) | 77.9 (33.0) | 0.28 |
|  | AG (3) | 56.7 (26.3) |  |
|  | AA (0) |  |  |
| rs2098948 | AA (40) | 77.7 (30.8) | 0.85 |
|  | AG (19) | 72.7 (36.1) |  |
|  | GG (5) | 74.8 (18.3) |  |
| rs4613543 | AA (63) | 77.0 (32.4) | 0.29 |
|  | AG (3) | 56.7 (26.3) |  |
|  | GG (0) |  |  |
| rs4496559 | CC (67) | 77.0 (32.9) | N/A |
| rs17060521 | AA (42) | 81.7 (35.3) | 0.34 |
|  | AG (22) | 70.3 (28.6) |  |
|  | GG (2) | 62.1 (12.1) |  |
| rs10520285 | TT (64) | 77.9 (33.0) | 0.28 |
|  | CT (3) | 56.7 (26.3) |  |
|  | CC (0) |  |  |
| rs2253270 | CC (66) | 76.9 (33.1) | N/A |
| rs2253170 | CC (66) | 77.0 (32.9) | N/A |
| rs13126570 | TT (42) | 81.7 (35.3) | 0.30 |
|  | CT (23) | 69.6 (28.1) |  |
|  | CC (2) | 62.1 (12.1) |  |
| rs34299544 | GG (39) | 82.2 (36.3) | 0.19 |
|  | CG (7) | 58.0 (11.5) |  |
|  | CC (15) | 73.1 (32.4) |  |
| rs2555661 | GG (66) | 76.0 (32.2) | N/A |
| rs6419992 | TT (40) | 80.2 (33.9) | 0.56 |
|  | CT (21) | 73.8 (34.3) |  |
|  | CC (6) | 66.5 (18.1) |  |
| rs17360116 | AA (64) | 77.9 (33.0) | 0.28 |
|  | AG (3) | 56.7 (26.3) |  |
|  | GG (0) |  |  |
| rs11724251 | AA (19) | 92.2 (34.8) | 0.037 |
|  | AG (30) | 73.8 (36.1) |  |
|  | GG (15) | 64.2 (15.3) |  |
| rs17060554 | TT (41) | 79.7 (33.1) | 0.34 |
|  | CT (21) | 68.4 (27.9) |  |
|  | CC (2) | 62.1 (12.1) |  |
| rs7349744 | GG (37) | 73.2 (31.9) | 0.071 |
|  | AG (26) | 76.8 (28.5) |  |
|  | AA (4) | 112.8 (53.6) |  |
| rs17060557 | TT (63) | 77.0 (32.4) | 0.29 |
|  | CT (3) | 56.7 (26.3) |  |
|  | CC (0) |  |  |
| rs2555630 | TT (66) | 76.0 (32.2) | N/A |
| rs2555631 | CC (66) | 76.0 (32.2) | N/A |
| rs3737012 | Failed QC |  |  |
| rs2254350 | GG (66) | 76.0 (32.2) | N/A |
| rs2612660 | AA (65) | 76.7 (33.1) | N/A |
| rs12644138 | CC (48) | 70.7 (29.5) | 0.0047 |
|  | CT (17) | 98.0 (34.6) |  |
|  | TT (2) | 49.0 (11.9) |  |
| rs12500316 | CC (40) | 77.4 (35.0) | 0.94 |
|  | CT (22) | 75.3 (26.8) |  |
|  | TT (3) | 71.4 (45.1) |  |
| rs17060596 | TT (49) | 75.4 (33.4) | 0.42 |
|  | CT (17) | 82.9 (32.0) |  |
|  | CC (0) |  |  |
| rs3797009 | TT (43) | 67.3 (26.7) | 0.0011 |
|  | CT (16) | 98.6 (35.0) |  |
|  | CC (2) | 49.0 (11.9) |  |
| rs1365614 | TT (63) | 74.1 (28.5) | N/A |
| rs1050145 | CC (27) | 84.3 (38.8) | 0.23 |
|  | CT (31) | 70.3 (25.1) |  |
|  | TT (8) | 70.5 (30.4) |  |
| rs1346270 | CC (66) | 76.6 (33.0) | N/A |
| rs6825010 | CC (64) | 76.7 (32.5) | N/A |
| rs1426945 | TT (19) | 72.6 (39.5) | 0.46 |
|  | CT (30) | 81.1 (31.7) |  |
|  | CC (14) | 69.0 (23.9) |  |
| rs12505520 | CC (66) | 76.0 (32.2) | N/A |
| rs3756273 | AA (20) | 72.1 (38.5) | 0.37 |
|  | AG (27) | 80.1 (29.5) |  |
|  | GG (12) | 65.2 (21.1) |  |
| rs4147098 | CC (24) | 76.4 (32.8) | 0.47 |
|  | CT (33) | 78.8 (33.8) |  |
|  | TT (7) | 62.0 (26.5) |  |
| rs1834693 | AA (31) | 75.4 (31.8) | 0.47 |
|  | AC (21) | 81.5 (37.8) |  |
|  | CC (2) | 51.8 (15.8) |  |
| rs13108146 | TT (39) | 75.4 (30.7) | 0.75 |
|  | GT (25) | 72.9 (31.7) |  |
|  | GG (0) | 118.9 (36.0) |  |
| rs17361009 | GG (56) | 74.0 (30.7) | 0.51 |
|  | GT (8) | 82.0 (42.6) |  |
|  | TT (0) |  |  |
| rs5007089 | TT (14) | 79.2 (31.2) | 0.79 |
|  | CT (40) | 78.0 (35.5) |  |
|  | CC (13) | 71.3 (27.3) |  |
| rs2255577 | GG (61) | 77.8 (33.1) | N/A |
| rs12645863 | GG (48) | 77.8 (35.4) | 0.65 |
|  | AG (15) | 74.5 (22.4) |  |
|  | AA (2) | 56.9 (4.6) |  |
| rs2251316 | CC (32) | 80.0 (36.7) | 0.89 |
|  | AC (23) | 75.5 (30.0) |  |
|  | AA (7) | 78.4 (26.7) |  |
| rs17295290 | TT (48) | 75.3 (32.3) | 0.90 |
|  | CT (14) | 78.5 (34.3) |  |
|  | CC (1) | 87.1 (-) |  |
| rs2555643 | GG (34) | 67.6 (28.7) | 0.11 |
|  | CG (28) | 84.6 (34.8) |  |
|  | CC (3) | 84.1 (35.0) |  |
| rs2555642 | TT (34) | 67.6 (28.7) | 0.057 |
|  | CT (30) | 86.9 (35.0) |  |
|  | CC (3) | 84.1 (35.0) |  |
| rs12643132 | GG (47) | 75.8 (33.1) | 0.69 |
|  | AG (15) | 74.5 (22.4) |  |
|  | AA (2) | 56.9 (4.6) |  |
| rs7657391 | CC (44) | 80.3 (32.5) | 0.52 |
|  | CT (21) | 72.2 (34.5) |  |
|  | TT (1) | 54.8 (-) |  |
| rs17361254 | TT (48) | 75.3 (32.3) | 0.52 |
|  | CT (15) | 76.4 (34.0) |  |
|  | CC (2) | 102.3 (21.6) |  |
| rs12644005 | GG (49) | 79.0 (36.1) | 0.61 |
|  | AG (15) | 74.5 (22.4) |  |
|  | AA (2) | 56.9 (4.6) |  |
| rs1346269 | TT (59) | 76.3 (31.7) | 0.65 |
|  | GT (8) | 82.0 (42.6) |  |
|  | GG (0) |  |  |
| rs17295603 | GG (59) | 76.3 (31.7) | 0.65 |
|  | AG (8) | 82.0 (42.6) |  |
|  | AA (0) |  |  |
| rs2555639 | TT (28) | 67.3 (29.4) | 0.076 |
|  | CT (33) | 80.0 (30.6) |  |
|  | CC (5) | 99.0 (47.1) |  |
| rs11725947 | AA (48) | 75.9 (33.2) | 0.54 |
|  | AG (14) | 74.9 (34.8) |  |
|  | GG (2) | 102.3 (21.6) |  |
| rs2256673 | TT (45) | 77.3 (33.4) | 0.58 |
|  | CT (15) | 76.4 (34.0) |  |
|  | CC (2) | 102.3 (21.6) |  |
| rs2256669 | AA (39) | 75.2 (29.9) | 0.41 |
|  | AC (26) | 81.6 (37.5) |  |
|  | CC (2) | 51.8 (15.8) |  |
| rs11133044 | CC (39) | 81.9 (37.1) | 0.34 |
|  | CG (24) | 70.8 (26.7) |  |
|  | GG (4) | 65.8 (6.6) |  |
| rs2555674 | CC (34) | 67.6 (28.7) | 0.057 |
|  | AC (30) | 86.9 (35.0) |  |
|  | AA (3) | 84.1 (35.0) |  |
| rs6811079 | GG (50) | 72.0 (30.7) | 0.020 |
|  | AG (16) | 93.9 (35.5) |  |
|  | AA (0) |  |  |
| rs2253442 | GG (30) | 67.5 (28.5) | 0.068 |
|  | AG (30) | 86.9 (35.0) |  |
|  | AA (2) | 84.1 (35.0) |  |
| rs2042755 | GG (22) | 82.9 (29.1) | 0.34 |
|  | AG (33) | 70.6 (31.5) |  |
|  | AA (10) | 81.2 (41.2) |  |
| rs2042756 | GG (22) | 82.9 (29.1) | 0.34 |
|  | CG (33) | 70.6 (31.5) |  |
|  | CC (10) | 81.2 (41.2) |  |
| rs1820526 | CC (39) | 81.9 (37.1) | 0.21 |
|  | CG (24) | 67.5 (22.6) |  |
|  | GG (3) | 67.7 (6.6) |  |
| rs1365625 | GG (22) | 82.6 (29.3) | 0.21 |
|  | GT (23) | 67.1 (28.6) |  |
|  | TT (10) | 82.6 (40.6) |  |
| rs2612677 | GG (37) | 74.0 (28.7) | 0.41 |
|  | AG (27) | 80.6 (37.1) |  |
|  | AA (2) | 51.8 (15.8) |  |
| rs17060632 | AA (53) | 77.3 (31.9) | 0.88 |
|  | AG (13) | 78.9 (37.1) |  |
|  | GG (0) |  |  |
| rs2877818 | CC (52) | 72.8 (30.9) | 0.11 |
|  | CT (14) | 88.2 (35.2) |  |
|  | TT (0) |  |  |
| rs10520286 | AA (53) | 77.3 (31.5) | 0.85 |
|  | AG (13) | 75.7 (34.9) |  |
|  | GG (0) |  |  |
| rs10019035 | CC (19) | 63.2 (17.7) | 0.055 |
|  | CT (6) | 80.4 (20.0) |  |
|  | TT (0) |  |  |
| rs10032848 | TT (24) | 72.8 (30.2) | 0.67 |
|  | GT (32) | 76.1 (31.2) |  |
|  | GG (10) | 83.8 (41.6) |  |
| rs10019203 | CC (24) | 72.8 (30.2) | 0.67 |
|  | AC (31) | 76.8 (31.4) |  |
|  | AA (10) | 83.8 (41.6) |  |
| rs9998916 | GG (50) | 73.9 (31.0) | 0.14 |
|  | CG (14) | 88.2 (35.2) |  |
|  | CC (0) |  |  |
| rs1978567 | Not available |  |  |
| rs17060646 | TT (24) | 72.8 (30.2) | 0.67 |
|  | CT (31) | 76.8 (31.4) |  |
|  | CC (10) | 83.8 (41.6) |  |
| rs10520281 | AA (52) | 72.8 (30.9) | 0.11 |
|  | AG (14) | 88.2 (35.2) |  |
|  | GG (0) |  |  |
| rs2332893 | GG (34) | 76.2 (30.9) | 0.45 |
|  | AG (24) | 81.9 (37.3) |  |
|  | AA (2) | 51.8 (15.8) |  |
| rs17362255 | AA (35) | 66.6 (28.1) | 0.058 |
|  | AC (27) | 85.4 (33.6) |  |
|  | CC (3) | 84.1 (35.0) |  |
| rs17296454 | CC (31) | 75.3 (30.1) | 0.60 |
|  | AC (31) | 78.6 (34.5) |  |
|  | AA (4) | 61.4 (34.6) |  |
| rs7657168 | AA (24) | 68.4 (24.7) | 0.28 |
|  | AG (40) | 81.9 (35.5) |  |
|  | GG (3) | 80.6 (51.1) |  |
| rs7687916 | TT (29) | 70.0 (22.4) | 0.33 |
|  | CT (30) | 79.5 (34.8) |  |
|  | CC (4) | 61.4 (34.6) |  |
| rs1365621 | TT (14) | 61.3 (24.2) | 0.20 |
|  | CT (37) | 77.1 (28.9) |  |
|  | CC (12) | 77.4 (32.2) |  |
| rs12646791 | CC (45) | 81.2 (37.5) | 0.13 |
|  | CT (20) | 67.5 (17.0) |  |
|  | TT (0) |  |  |
| rs17362542 | GG (50) | 78.7 (30.6) | 0.48 |
|  | AG (16) | 69.9 (39.8) |  |
|  | AA (1) | 102.3 (-) |  |
| rs3113888 | TT (17) | 65.9 (31.7) | 0.44 |
|  | AT (38) | 77.4 (31.0) |  |
|  | AA (7) | 74.6 (22.3) |  |

*p-value of ANOVA for differences in gene expression by SNP genotype
